# Entomological indicators and food sources of triatomines in the Brazilian semi-arid region

**DOI:** 10.1590/0037-8682-0573-2023

**Published:** 2024-08-16

**Authors:** Luis Ricardo Soares da Silva, João Paulo Sales Oliveira-Correia, Francisco José de Freitas Araújo, Cleber Galvão, Maria Beatriz Araújo Silva, Jaqueline Bianque de Oliveira

**Affiliations:** 1 Universidade Federal Rural de Pernambuco, Programa de Pós-graduação em Biociência Animal, Recife, PE, Brasil.; 2 Instituto Oswaldo Cruz, Fiocruz, Laboratório Nacional e Internacional de Referência em Taxonomia de Triatomíneos, Rio de Janeiro, RJ, Brasil.; 3 Secretaria Municipal de Saúde de Petrolina, Petrolina, PE, Brasil.; 4 Universidade de Pernambuco, Faculdade de Enfermagem Nossa Senhora das Graças, Recife, PE, Brasil.; 5 Secretaria Estadual de Saúde de Pernambuco, Laboratório Central de Saúde Pública Dr. Milton Bezerra Sobral, Recife, PE, Brasil.; 6 Universidade Federal Rural de Pernambuco, Laboratório de Parasitologia, Recife, PE, Brasil.

**Keywords:** Vectors, Household infestation index, Natural infection index, Disease ecology, Epidemiology, One Health

## Abstract

**Background:**

Triatomines are biological vectors of *Trypanosoma cruzi*, the etiological agent of Chagas Disease (CD) and have various mammalian hosts. This study evaluated the entomological indicators and food sources of triatomines in Petrolina in the semi-arid region of Brazil, where CD is endemic.

**Methods:**

Triatomines were captured indoors and outdoors through an active search and entomological indices (household and natural infections) were calculated. Parasitological analyses were performed through microscopic visualization using Giemsa-stained insect feces, and DNA sequencing was employed to identify food sources from the gut contents of 82 insects (9.05%) that were better preserved.

**Results:**

We captured triatomines (906) in peridomicile (807) and intradomicile (99): *Triatoma brasiliensis* (84.7%, 767 specimens), *Triatoma* spp. (8.2%, 74 specimens), *T. pseudomaculata* (6.5%, 59 specimens), *Rhodnius* spp. (0.4%, four specimens), *R. nasutus* (0.1%, one specimen), and *T. sordida* (0.1%, one specimen). The household infestation index is 11.8%. Thirty-five triatomines were infected (33 *T. brasiliensis* and two *T. pseudomaculata*), corresponding to a natural infection index of 3.8%. The identified food sources were human *T. pseudomaculata* and *T. brasiliensis*, dogs for *T. brasiliensis* and rodents (*Mus musculus*) for *T. brasiliensis*.

**Conclusions:**

The results reinforce the need to intensify CD diagnosis, surveillance, and control actions, as an increase in entomological indices was recorded. Blood from humans and domestic and synanthropic animals was detected in the infected triatomines, suggesting a risk of CD vector transmission in Petrolina. As CD is a zoonosis, multidisciplinary and intersectoral CD surveillance must be conducted in the context of the One Health.

## INTRODUCTION

Triatomines (Hemiptera, Triatominae) are vectors of *Trypanosoma cruzi* (Chagas, 1909) (Kinetoplastida, Trypanosomatidae), a protozoan that causes Chagas Disease (CD)^1^. Neglected tropical diseases are endemic in 21 countries in Latin America, with approximately 6-8 million infected individuals, 30,000 cases per year and 10,000 deaths^2^. Transmission also occurs through ingestion of food contaminated with *T. cruzi* ^3^. 

Triatomines are found in domestic, peridomestic, and wild environments^4^, and 64 species of Hemiptera have been identified in Brazil^5^ ^-^ ^6^. In Pernambuco, 14 species have already been recorded^7^, namely: *Panstrongylus geniculatus* (Latreille, 1811), *Panstrongylus megistus* (Burmeister, 1835), *Panstrongylus lutzi* (Neiva & Pinto, 1923)*, Panstrongylus tibiamaculatus* (Pinto, 1926)*, Rhodnius nasutus* Stål, 1859, *Rhodnius neglectus* Lent, 1954, *Triatoma brasiliensis* Neiva, 1911, *Triatoma infestans* (Klug, 1834), *Triatoma melanocephala* Neiva & Pinto, 1923, *Triatoma pseudomaculata* Corrêa & Espínola, 1964, *Triatoma petrocchiae* Pinto & Barreto, 1925, *Triatoma rubrofasciata* (De Geer, 1773), *Triatoma sordida* (Stål, 1859), and *Psammolestes tertius* Lent & Jurberg, 1965^8^ ^-^ ^11^. *T. brasiliensis* and *T. pseudomaculata* are the most relevant species throughout northeastern Brazil^6^ ^,^ ^12^ ^-^ ^13^ as they have a high capacity for adaptation to intra-domestic, peridomestic, and wild environments, although *T. brasiliensis* presents superior vector capacity and competence^13^.

The transmission of *T. cruzi* occurs in three cycles: (i) wild, which involves triatomines and wild mammals; (ii) peridomestic animals, including domestic animals (dogs, cats, pigs, and goats), synanthropic animals (such as marsupials and rodents), and wild triatomines, which are attracted to food sources inside homes; and (iii) domestic animals, which are related to transmission between humans, domestic animals, and triatomines^14^ ^-^ ^16^.

Mammals from different orders act as hosts for *T. cruzi* and those that stand out in the morphoclimatic domain of the Caatingas, in the Brazilian semi-arid region^17^ ^-^ ^18^. Dogs act as sentinels to identify areas at risk of CD emergence, as highlighted in studies in Argentina^19^, Brazil^20^, and the United States^21^. These mammals are considered one of the primary elements of transmission within the home, serving as a bridge between wild and domestic cycle^16^ ^,^ ^22^, in addition to being susceptible to manifesting the disease^19^ ^,^ ^21^ ^,^ ^23^.

Among CD surveillance and control strategies, some entomological indicators stand out, such as the household infestation index, related to the index of household units with the occurrence of triatomines, and the natural infection index, associated with the percentage of triatomines positive for trypanosomatids^24^.

Pernambuco is at risk vector transmission of CD^25^. From 2012 to 2017, Petrolina was the municipality in the Sertão region of the São Francisco River that had the largest number of triatomines captured as well as the largest number of insects infected by *T. cruzi* ^26^. The study of triatomine food sources can significantly support the knowledge about their natural hosts and their role in the transmission of *T. cruzi*, effectively contributing to the epidemiological surveillance of the disease. According to the Coordenação Municipal de Controle da Doença de Chagas de Petrolina, in the last 4 years, 39 cases of chronic CD have been diagnosed in the municipality of Petrolina due to vector transmission^26^. Therefore, this study aimed to understand the entomological indicators and identify the hosts that act as food sources for triatomines in the municipality of Petrolina, to contribute to CD surveillance and control actions in Pernambuco from the perspective of the One Health.

## METHODS

### ● Study area

**FIGURE 1:**
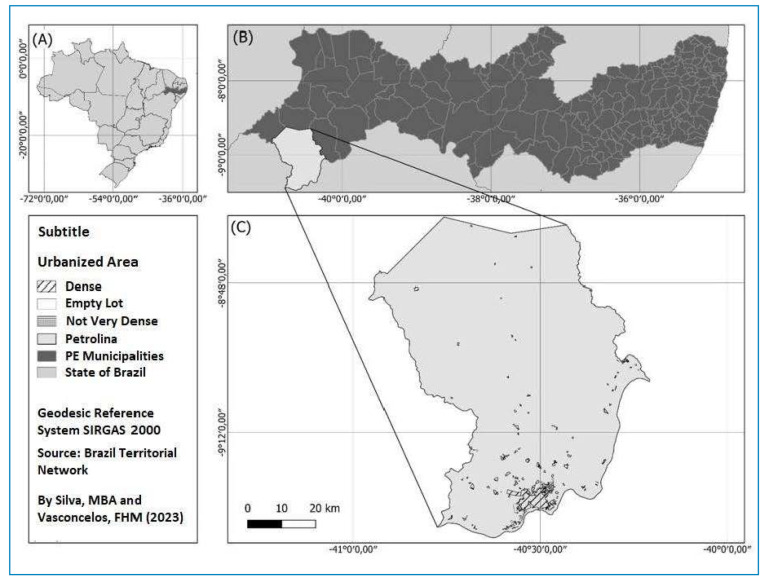
Map of study area, Petrolina, Pernambuco state, Brazil.

The municipality of Petrolina (9º 23' 55'' S, 40º 30' 3'' W) is in the morphoclimatic domain of the Caatingas^27^, in the semi-arid region of Pernambuco, in the Sertão of São Francisco River region^28^ ^,^ ^29^ (Figure 1). It has the third largest population in the state of Pernambuco: 386,786 inhabitants, with a demographic density of 84.79 inhabitants/km2, an area of 4,561.870 km2; 72.7% of households with adequate sanitation, 91.9% of urban homes on tree-lined public roads, and 8.7% of homes with adequate urbanization^30^. The average annual rainfall is 560 mm, temperature with average variations between 24 ºC and 28 ºC and average relative humidity between 66% and 72% in the wettest months^31^.

### ● Ethical considerations, collection, and processing of triatomines

The insects were captured from January to December 2022, during entomological research carried out by health agent of the municipality, following the routine of the State Chagas Disease Surveillance and Control Program (PCDCh), in accordance with the “Manual of Surveillance, Prevention and Control of Zoonoses: technical and operational standards” of the Ministry of Health^32^ and the guidelines of Technical Note 36/2012 of July 25, 2012, of the Ministry of Health. This is the standard procedure used in entomological research to control CD in Pernambuco. Therefore, it was not necessary to submit this research to the Human Research Ethics Committee.

Insects were collected using entomological tweezers and flashlights to inspect crevices and places without lighting, and all rooms in the household units were inspected. Entomological research began in houses/intradomiciles (walls, furniture, and mattresses). Subsequently, inspections were conducted in the vicinity/peridomicile (grain and tool storage areas, chicken coops, animal pens, stone walls, fences, and rubble).

All the activities and pertinent data were recorded in a specific form and used for routine entomological research. The presence of a single triatomine specimen was sufficient for the household unit to be considered positive in entomological research, intra-domicile, and/or peridomicile. The triatomines were sent to the Regional Entomology Laboratory of VIII GERES and the Pernambuco Central Laboratory Dr. Milton Bezerra Sobral - LACEN/Central Laboratory of Endemics (LABEND) for taxonomic identification and parasitological research. Specimens with incomplete data on their origin, those without specific identification, and those without conditions for examination were excluded. The specimens were identified to the species level according to Galvão and Gurgel-Gonçalves^10^, Lent and Wygodzinsky^33^, and Gurgel-Gonçalves et al.^34^. The identification of *Triatoma brasiliensis* nymphs was based on the following morphological characteristics: tibiae with a subapical ring, a pronotum design that looks like two question marks, and the dorsal surface of the abdomen without a defined design^35^.

The household infestation index was calculated using the following formula^24^: household infestation index = (total number of household units with triatomines/total number of household units surveyed) × 100.

For parasitological analyses using the conventional method in triatomines, fresh intestinal contents of the insects were examined by abdominal compression. All captured insects were examined and confirmed through visualization of the parasites by direct examination of triatomine feces and subsequent confirmation of the stained slides using GIEMSA^36^. The natural infection index was calculated using the following formula^24^: natural infection index = (total number of infected triatomines/total number of insects examined) × 100.

### ● Identification of triatomine food sources

Not all captured triatomines were processed to identify the food sources. The best preserved triatomines infected with *T. cruzi* were used, from which intestinal contents were used for DNA extraction. Intestinal content samples were processed at the National and International Reference Laboratory for Triatomine Taxonomy of the Oswaldo Cruz Institute Foundation to identify food sources.

Genomic DNA was extracted using a DNeasy Blood & Tissue kit (Qiagen) following the manufacturer's protocol. DNA concentration was estimated using the Quantus TM Fluorometer (Promega). The PCR mix without DNA was used as a negative control, and triatomine specimens from the National and International Reference Laboratory for Triatomine Taxonomy Insectary (with controlled feeding) were used as positive controls.

To identify the food source, PCR was performed using the Platinum II Hot-Star Master Mix Kit (Thermo Fisher Scientific) and the 12S rDNA marker^37^ ^-^ ^38^. The PCR products were electrophoresed on an agarose gel (2%), stained with Gel Red (Biotium, Inc., California, USA), and observed under UV light. The amplicons were purified using EXOSAP (Affymetrix, USA).

DNA fragments were sequenced by Sanger sequencing. Sequences were visualized, edited, and aligned using Bioedit v. 7.0.5 (Department of Microbiology, North Carolina State University) and Lasergene SeqMan TM v. 7 (DNStar, Madison, Wisconsin, USA) and compared using Blast (Basic Local Alignment Search Tool) in the National Center for Biotechnology Information database (http://blast.ncbi.nim.nih.gov/Blast.cgi). Only sequences with a coverage above 95% were considered for species identification.

### ● Data analysis

Data were analyzed using descriptive statistics. The chi-square test (χ²) was used to analyze the number of triatomines captured at the intradomicile and peridomicile, and the G-test was used to compare triatomine species with the capture environment (intradomicile or peridomicile). Statistical significance was set at 5%. The BioEstat software version 5.3 was used.

## RESULTS

According to secondary data provided by the Petrolina Municipal Health Department, 906 triatomines will be captured in the municipality in 2022. *Triatoma brasiliensis* accounted for 84.7% (767/906) of the total (419 nymphs and 348 adults), *Triatoma* spp. accounted for 8.2% (74/906 nymphs), and *T. pseudomaculata* 6.5% (59/906 adults). *Rhodnius* spp. constituted 0.4% (4/906 nymphs) of the total, *R. nasutus* represented 0.1% (1/906 adults), and *T. sordida* 0.1% (1/906 adults) (Table 1).

**TABLE 1:** Triatomine species and natural infection index by *Trypanosoma cruzi* in the municipality of Petrolina, an endemic area of Chagas Disease in Brazilian semi-arid region.

Species	Peridomicile								Intradomicile								Total		
	Adults (n)	%	FS (n)	Nymphs (n)	%	FS (n)	Total	%	Adults (n)	%	FS (n)	Nymphs (n)	%	FS (n)	Total	%	Total	%	IIN
*Triatoma brasiliensis*	303	44.8	31	373	55.2	20	676	83.8	45	49.5	10	46	50.5	11	91	91.9	767	84.7	4,3
*Triatoma* spp.	0	0	0	70	0	0	70	8.7	0	0	0	4	100	0	4	4	74	8.2	0
*Triatoma pseudomaculata*	56	100	9	0	0	0	56	6.9	3	100	1	0	0	0	3	3	59	6.5	3,4
*Rhodnius* spp.	0	0	0	4	100	0	4	0.5	0	0	0	0	0	0	0	0	4	0.4	0
*Rhodnius nasutus*	1	100	0	0	0	0	1	0.1	0	0	0	0	0	0	0	0	1	0.1	0
*Triatoma sordida*	0	0	0	0	0	0	0	0	1	100	0	0	0	0	1	1	1	0.1	0
**Total**	**360**	**44.6**	**40**	**447**	**55.4**	**20**	**807**	**89.1**	**49**	**49.5**	**11**	**50**	**50.5**	**11**	**99**	**10.9**	**906**	**100**	**3,8**

Legend: **FS**: Used for the analysis of food sources; **IIN**: natural infection index.

Overall, 2,717 household units were visited, with a household infestation index of 11.04% (300/2717).

Of the 906 triatomines captured, 807 (89.1%) were captured in peridomicile and 99 (10.9%) in intradomicile (χ² = 313.39, df = 4, p = 1.40 E-66). The predominant species in the peridomicile area was *T. brasiliensis* (676), followed by *Triatoma* spp. (70), *T. pseudomaculata* (56), *Rhodnius* spp. (4), and *R. nasutus* (1). In the intradomicile period, this scenario was also repeated, with *T. brasiliensis* being the most frequent (91), followed by *Triatoma* spp. (4), *T. pseudomaculata* (3), and *T. sordida* (1) (G-test = 7.1319; p = 0.0283) (Table 1).

Of the 906 captured insects, 35 were infected, corresponding to a natural infection index of 3.8%. Regarding *T. brasiliensis*, 33 specimens were infected, with a natural infection index of 4.3%, whereas two specimens of *T. pseudomaculata* were infected, representing a natural infection index of 3.4%.

To identify food sources, 82 triatomine specimens were used: *T. brasiliensis* (72 specimens: 41 adults and 31 nymphs) and *T. pseudomaculata* (10 adult specimens). Regarding the location of capture, 60 (51 *T. brasiliensis* and nine *T. pseudomaculata*) were in the peridomicile (animal pens, chicken coops, stone walls, fences, and rubble) and 22 (21 *T. brasiliensis* and one *T. pseudomaculata*). Of the 82 triatomines analyzed, 11 were infected: nine with *T. brasiliensis* and two with *T. pseudomaculata*, which corresponded to a natural infection index of 13.4% (Table **1**).

Of the 82 specimens, in five (one nymph and four adults), it was possible to identify the food source: human blood in *T. pseudomaculata* (two) and *T. brasiliensis* (one), dog blood in *T. brasiliensis* (one), and rodent (*Mus musculus*) blood in *T. brasiliensis* (one) (Table 2). All five specimens whose food sources were identified were infected. Of the three triatomines in which human blood was identified, two were intra-domicile and one was peridomicile, whereas the two triatomines infected with dog and rodent blood were captured in the peridomicile (Table 2).

**TABLE 2:** Species of infected triatomines captured in the municipality of Petrolina, an endemic area of Chagas Disease in Brazilian semi-arid, according to the stage of development and identified food source.

Triatomines	Capture location	Estage of development	Food source
*Triatoma pseudomaculata*	intradomicile	adult	*Homo sapiens*
*Triatoma brasiliensis*	intradomicile	adult	*Homo sapiens*
*Triatoma brasiliensis*	peridomicile	adult	*Canis lupus familiaris*
*Triatoma pseudomaculata*	peridomicile	adult	*Homo sapiens*
*Triatoma brasiliensis*	peridomicile	nymph	*Mus musculus*

## DISCUSSION

According to Jansen et al.^16^, the assessment of infection risks for humans in each location must jointly consider the triatomine fauna, mammalian hosts, and reservoirs. In this regard, identification of the food sources of triatomines is an important contribution to the knowledge of their natural hosts and their respective roles in the transmission of *T. cruzi*, effectively contributing to the epidemiological surveillance of the disease, mainly using molecular techniques. This study is pioneering in Pernambuco in terms of the molecular identification of food sources for infected triatomines.

*T. brasiliensis* and *T. pseudomaculata* are triatomines adapted to the morphoclimatic domain of the Caatingas, and a semi-arid climate has been identified in previous studies conducted in Petrolina from 2012 to 2017^13^ ^,^ ^39^. Both are of epidemiological importance because of their high capacity to adapt to intradomicile, peridomicile, and wild environments, although *T. brasiliensis* has superior vector capacity and competence^11^.

According to the Coordenação Municipal de Controle da Doença de Chagas de Petrolina^26^, the household infestation index in the present study (11.04%) was higher than that in 2018 (5.5%). The increase in this rate may have occurred because of the suspension of visits by Endemic Disease Control Agents (ACE) during the COVID-19 pandemic. Therefore, spraying aimed at eliminating triatomines from the home environment was not performed in this study. Alpha-cypermethrin has been used in Pernambuco for two decades^40^.

The household infestation index in Petrolina was higher than that of the entire state of Pernambuco in recent years: 2007 (9.45%), 2008 (8.9%), 2009 (8.8%), 2010 (9, 0%), 2011 (7.1%), 2012 (6.9%), 2013 (8.6%), 2014 (7.7%), 2015 (8.3%), 2016 (8.5%), 2017 (6.7%)^41^ ^-^ ^42^. From 2011 onward, surveillance actions were implemented in the state through the Program to Combat Neglected Diseases (SANAR), which were directly reflected in the household infestation index. The state no longer had numbers higher than before the implementation of the SANAR, which established the goal of maintaining the household infestation index in municipalities below 10%^41^ ^-^ ^42^.

The natural infection index of Petrolina in the present study (3.8%) was higher than that in the period 2012-2017 (2.4%)^13^. *T. brasiliensis* had a natural infection index (4.3%) higher than that in 2018 in the municipality (3.8%); in 2017, the natural infection index of *T. brasiliensis* was also lower (2%)^13^, while in 2016, no infected triatomines were found in the municipality^23^. The natural infection rate of this species exhibited a potentially unstable pattern: 5.5% in 2012, 2.1% in 2013, 1.6% in 2014, and 6% in 2015^13^. No statistical tests were performed to validate the observed fluctuations.

The results of the present study are in line with those of other studies conducted in Pernambuco regarding the natural infection index of *T. cruzi* in *T. brasiliensis*: 6.5%^40^, 19.2%^39^, 7.41%^43^, 10.50%^36^, and 12,2%^44^. In other studies, carried out in the northeastern region, *T. brasiliensis* was also found infected in Piauí (7.18%)^34^ and in Rio Grande do Norte (1.2%)^45^ (2.8%)^46^.

*Triatoma pseudomaculata* had a lower natural infection rate (3.4%) than in 2017 in the municipality (1.4%)^13^. Notably, no infected specimens were observed between 2013 and 2016^10^. In 2012, the infection rate of *natural T. pseudomaculata* was 7.7%^26^. Similar to the present study, *T. pseudomaculata* presented with infection in other studies conducted in Pernambuco: 8%^40^, 13.1%^39^, 8.70%^36^, and 12.90%^43^. Research carried out in northeastern Brazil confirms *T. cruzi* infection in *T. pseudomaculata*: Piauí (2.28%)^34^, Rio Grande do Norte (0.1%)^44^ and Bahia (4%)^47^. Outside the northeastern region, infection was detected in *T. pseudomaculata* in Goiás in a study that did not record the infection rate^47^.

In the present study, infection by *T. cruzi* was not detected in *T. sordida*, which was also recorded by Silva et al.^13^ not only in Petrolina, but also in all municipalities in the Sertão of the São Francisco River region from 2012 to 2017. Silva et al.^40^, Silva et al.^43^, and Medeiros et al.^36^ also did not record infections in this species of triatomine in Pernambuco. In contrast, in Goiás^48^, Paraná^49^, and Bahia^47^ *T. cruzi* infection was recorded in *T. sordida*.

Infection with *R. nasutus* was also not detected in the present study, which has also been reported in the state^40^ and Rio Grande do Norte^45^. This result differed from those obtained by Piauí^34^ and Pernambuco^36^.

The municipality of Petrolina demonstrates a certain seasonality of species, except *T. brasiliensis* and *T. pseudomaculata*, which were frequently recorded. From 2012 to 2017, *T. sordida* was recorded from 2013 to 2017^13^, while *R. nasutus* was recorded only in 2018^13^. 

In the present study, it was possible to identify food sources for only 6% of the triatomines studied. One factor that may have prevented DNA amplification was the preservation of the captured material before DNA extraction. It is also important to consider that among the Brazilian morphoclimatic domains, the Caatinga has one of the highest temperatures, especially during the dry period, which accelerates the digestion of blood consumed by triatomines^50^.

In a study carried out in Pernambuco, using the precipitin technique, Silva et al.^51^ identified the eclectic eating behavior of *T. brasiliensis* and *T. pseudomaculata* when recording birds, rodents, dogs, opossums, lizards, cattle, goats, cockroaches, pigs, and humans as food sources. Using PCR, rodents from four families were identified as the most frequent food sources of *T. brasiliensis* in the municipality of Tauá, Ceará^52^. 

In the present study, *T. brasiliensis* and *T. pseudomaculata* were the most common triatomines, and hematophagy was observed in humans, dogs, and rodents. Similar PCR results were obtained in the states of Paraíba and Rio Grande do Norte^53^. However, unlike the present study, in which human blood was the most frequent food source, dog blood was the main food source for triatomines, followed by humans and rodents such as *Mus musculus* ^53^.

Similar to other rodents^17^, in the present study, the house mouse (*M. musculus*) was identified as a food source for *T. brasiliensis*, which was also reported by Honorato et al.^53^ using PCR. This rodent species, which was introduced in Brazil, may have a relevant epidemiological role, either as a host for triatomines or as a reservoir for *T. cruzi*. This rodent is generally associated with human habitation and is considered a generalist synanthropic species^54^, presenting a cosmopolitan distribution and being responsible for most of the damage caused to the economy and public health^55^. When these animals access their homes, infected triatomines can feed on them, facilitating the transmission of *T. cruzi* to both humans and domestic animals.

Similar to the results of the present study, Ribeiro et al.^56^ used PCR to identify human and dog blood in *T. brasiliensis* and *T. pseudomaculata* in the dog blood in Bahia. Similar to the present study, the vast majority of triatomines have been captured in an intradomicile environment.

The detection of human blood as a food source in triatomines of *T. brasiliensis* and *T. pseudomaculata* captured intradomiciles is of epidemiological importance. It is important to highlight that *T. brasiliensis* is more anthropophilic than *T. pseudomaculata* ^7^. 

Pernambuco State is at risk for vector transmission of CD, and Petrolina was the municipality in the Sertão of the São Francisco River that, from 2012 to 2017, had the largest number of triatomines captured, as well as the largest quantity of infected insects^13^. According to the Coordenação Municipal de Controle da Doença de Chagas de Petrolina^26^, 39 cases of chronic CD. All cases were related to vector transmission. 

The presence of nymphs of *T. brasiliensis* and *Triatoma* spp. in the intra-domicile region is traditionally used as a parameter for vector control. This is a sign of adaptation of the species to the artificial ecotope, as nymphs do not have wings^45^ ^,^ ^57^.

The majority of insects were captured in the peridomicile because of the presence of domestic animals such as dogs, cats, pigs, sheep, cattle, horses, goats, and birds, as well as synanthropic animals such as rodents^17^ ^-^ ^18^. The peridomicile is considered a link between wild and domestic cycles^15^ ^,^ ^52^ ^,^ ^57^, in which dogs play a prominent role^15^ ^-^ ^16^. Dogs can act as sentinels to identify areas at risk of CD emergence, in addition to being susceptible to manifesting the disease^19^ ^-^ ^21^ ^,^ ^23^. Studies conducted in dogs have demonstrated a high seroprevalence of anti-*T. cruzi*, which demonstrates the exposure of these animals to the protozoan and confirms their status as sentinel host^20^ ^,^ ^58^. Furthermore, compared to humans, dogs are more susceptible as hosts of triatomines and are consequently infected with *T. cruzi* ^59^. 

In Pernambuco and other states where CD is endemic, the role of dogs as hosts of *T. cruzi* and other animals susceptible to the disease must be investigated. In the present study, the detection of human, dog, and rodent blood in triatomines demonstrates the risk of vector transmission of *T. cruzi* in the studied region.

*T. brasiliensis* occurs exclusively in Brazil^18^ and is considered the most important vector in the northeastern region^7^ because it presents some epidemiologically important characteristics, such as being the most frequent species, having a wider geographic distribution^51^, a higher rate of natural infection by *T. cruzi* and a higher level of anthropophilia^7^. *T. pseudomaculata* stands out as the second most important species in the transmission of *T. cruzi* ^7^ ^,^ ^43^, as it is found both indoors and outdoors, in domestic animal breeding facilities, and in fences made of dry twigs associated with *Cereus jamacaru* (Mandacaru), in addition to wild environments, such as marsupial and rodent burrows^8^ ^-^ ^9^.

According to Jansen et al.^16^, “*due to the multiplicity of T. cruzi vectors and hosts, CD should not be seen solely as a human parasitic disease*”. Therefore, for the effective control of this neglected tropical disease, multi-professional, transdisciplinary, and intersectoral actions are necessary, as recommended by the One Health approach, as this disease is associated with ecological aspects inherent to the human-animal-vector-ecosystem interaction^16^ ^,^ ^60^.

These results reinforce the need to intensify CD diagnosis, surveillance, and control actions in Petrolina, as an increase in entomological indices was recorded in addition to the detection of the blood of humans and domestic and synanthropic animals as a food source for infected triatomines, suggesting the risk of vector transmission of CD in the municipality. Multidisciplinary and intersectoral actions must be performed in the context of the One Health approach.
